# Maternal attachment state of mind and defensive functioning in pregnancy: predicting mother–infant relationship at 6 months through the PDM-2 Infancy and Early Childhood section

**DOI:** 10.3389/fpsyg.2025.1568620

**Published:** 2025-04-04

**Authors:** Nicola Carone, Jacopo Tracchegiani, Vittorio Lingiardi

**Affiliations:** ^1^Department of Systems Medicine, University of Rome Tor Vergata, Rome, Italy; ^2^Department of Brain and Behavioral Sciences, University of Pavia, Pavia, Italy; ^3^Department of Dynamic and Clinical Psychology, and Health Studies, Sapienza University of Rome, Rome, Italy

**Keywords:** attachment, coherence of mind, defensive functioning, mother–infant relationship, *Psychodynamic Diagnostic Manual (second edition; PDM-2)*

## Abstract

**Background:**

Pregnancy is a period of profound psychological reorganization, marked by increased vulnerability and the reactivation of past attachment experiences. During this transition, maternal attachment states of mind and the ability to regulate emotional distress through unconscious defenses play a crucial role in shaping early mother–infant relationships. Although the *Psychodynamic Diagnostic Manual, second edition* (PDM-2) was not designed as a parenting manual, it provides a valuable framework for assessing how maternal attachment and defensive functioning influence the mother–infant relationship, since it emphasizes defensive functioning as a core component of mental functioning.

**Aims:**

This longitudinal study examines the relationship between maternal attachment mental states during the third trimester and mother–infant relationship quality at 6 months postpartum, while also exploring the mediating role of maternal defensive functioning.

**Methods:**

A community sample of 68 cisgender heterosexual pregnant women (*M_age_* = 35.83 years; *SD* = 4.32) was recruited during the third trimester (Time 1 [T1]; *M_gestational age_* = 36.30 weeks, *SD* = 1.80). At T1, maternal attachment coherence of mind was assessed using the Adult Attachment Interview (AAI), and defensive functioning was evaluated applying the Defense Mechanism Rating Scale–Q sort to AAI transcripts. At 6 months postpartum (Time 2 [T2]), the mother–infant relationship quality was assessed using the Infant–Caregiver Relationship Scale, as detailed in Section IV of the Psychodiagnostic Chart—Infancy and Early Childhood from the PDM-2. At T2, 58.82% of infants were assigned female at birth, with a mean age of 6.04 months (*SD* = 0.34). All participating mothers resided in Italy and were partnered with their infants’ fathers.

**Results:**

Mediation analysis showed that greater prenatal attachment coherence of mind was directly associated with better mother–infant relationship quality. Furthermore, mother–infant dyads in which mothers exhibited lower AAI coherence of mind demonstrated poorer relationship quality via less adaptive maternal defensive functioning.

**Conclusion:**

Maternal attachment mental states and defensive functioning during pregnancy play critical roles in the development of early caregiving relationships. The findings also highlight the PDM-2’s relevance in understanding maternal mental functioning and emphasize the need for targeted parenting interventions during pregnancy and early postpartum.

## 1 Introduction

Pregnancy represents a unique developmental phase and a period of profound psychological reorganization in a woman’s life, marked by heightened vulnerability and the reactivation of past attachment experiences and relational patterns (Behringer et al., 2011; Stern, 1995). This transitional phase significantly influences the emerging mother–child relationship, with long-term implications for child development (Slade et al., 2009) across multiple domains, including emotional regulation, cognitive abilities, and social functioning (e.g., Branjerdporn et al., 2017; Claridge, 2021). During pregnancy, maternal internal working models of attachment relationships become especially salient, as mothers anticipate and emotionally prepare for caregiving (George and Solomon, 2008). Mothers bring their internal working models of relationships, shaped by early attachment experiences (Bowlby, 1980), into their role as caregivers. Then, these models guide their expectations, emotions, and behaviors in their interactions with children (Main et al., 1985).

Of note, internal working models of attachment should be distinguished from attachment styles, which social psychologists use to describe an adult’s self-reported attitudes toward close relationships (Shaver and Mikulincer, 2002). In this context, self-report measures of attachment are generally understood to assess cognitive schemas related to the perceived availability of others in close relationships, rather than providing a direct window into unconscious attachment-related processes (Shaver and Mikulincer, 2002; Strauss et al., 2022). This distinction is further supported by the weak associations observed between attachment styles and states of mind regarding attachment (Roisman et al., 2007).

Research has shown that a mother’s attachment state of mind during pregnancy is a robust predictor of her infant’s later attachment pattern (Fonagy et al., 1991; Jacobvitz et al., 2025; Madigan et al., 2015; van IJzendoorn, 1995). Mothers classified as secure-autonomous—characterized by narrative coherence, emotional accessibility, and high reflective functioning—are more likely to foster secure attachment in their infants (e.g., Verhage et al., 2016). These mothers also tend to exhibit higher sensitivity to their children’s cues, which is an essential component of effective caregiving (e.g., De Wolff and van IJzendoorn, 1997; Madigan et al., 2024). Conversely, mothers with insecure or disorganized attachment patterns often struggle with emotional availability and consistency in caregiving (e.g., Jacobvitz et al., 2025; Lyons-Ruth et al., 1999). Insecure maternal attachment is reflected in dismissive or entangled/preoccupied states of mind, which may result in caregiving that is emotionally withdrawn, intrusive, or inconsistent (Madigan et al., 2006). Finally, mothers with a disorganized attachment mental state, often rooted in unresolved trauma or loss, stand at particularly high risk for difficulties in caregiving, as they are more likely to experience their infants’ needs as overwhelming or threatening (Lyons-Ruth et al., 1999).

Pregnancy may also trigger a distinct set of psychological stressors, including fears about childbirth, concerns regarding caregiving competence, and the potential reactivation of unresolved relational traumas (Behringer et al., 2011; Slade et al., 2009), which may elicit defenses—unconscious strategies for managing internal conflicts and regulating emotional distress (Cramer, 2006; Vaillant, 1977, 1992). Adaptive defenses, such as suppression or humor, may help mothers manage stress while remaining emotionally available to their infants (Perry, 2016). For example, a mother who suppresses minor anxieties about childbirth may remain focused on bonding with her baby, thereby fostering a positive relational environment (Porcerelli et al., 2016, 2022). In contrast, maladaptive defenses, such as projection, denial, or dissociation, may impede caregiving by distorting mothers’ perceptions of her infant or diminishing her capacity for emotional regulation (Perry, 2016). A mother who relies on projection, for instance, may misinterpret her infant’s cues as evidence of rejection or hostility, thereby disrupting the caregiving bond (Carone, 2025). Moreover, unresolved trauma or loss, often accompanied by maladaptive defensive strategies, increases the risk of inconsistent or emotionally unavailable maternal behaviors (Hesse and Main, 2000; Lyons-Ruth et al., 1999).

Of particular relevance to the present study, research has shown that, by 6 months postpartum, the mother–infant relationship has begun to solidify, demonstrating reciprocal interactions and emerging attachment behavior in the infant (Ainsworth et al., 1978). This critical period establishes the dyadic foundation upon which subsequent developmental milestones are built. Both pre-existing maternal factors and the evolving dynamics of the dyad strongly influence the quality of maternal caregiving during these early months (George and Solomon, 2008; Slade et al., 2009). Similarly, a mother’s way of managing intrapsychic challenges through her defensive repertoire has direct implications for the quality of her postnatal relationship with her infant (Perry, 2016). In this vein, secure and insecure attachment states of mind may shape the types of defenses a mother is likely to employ (Carone et al., under review; Porcerelli et al., 2016, 2022).

Previous research on self-reported adult attachment in non-parenting contexts (Fraley et al., 1998; Mikulincer and Horesh, 1999) has highlighted distinct defensive patterns associated with self-reported insecure attachment orientations. Avoidant individuals, in contrast to secure individuals, are prone to idealization (i.e., viewing caregivers and their childhood experiences more positively than is supported by specific memories or details). They may also rely on projective mechanisms (i.e., perceiving denied aspects of the self in others). Similarly, avoidant individuals have been found to favor repressive defenses (Fraley and Shaver, 1997; Mikulincer and Orbach, 1995). Conversely, anxious-ambivalent individuals have been shown to make greater use of projective-identification, perceiving others through the lens of their own self-descriptions and denying separation from significant others (Fraley et al., 1998; Mikulincer and Horesh, 1999).

Of note, these findings are limited by their reliance on self-report questionnaires, which may present greater bias (relative to observer-based measures) in their assessment of predominantly unconscious processes, such as attachment states of mind and defenses. In fact, self-report and observer-rated measures of attachment [e.g., the Adult Attachment Interview (AAI)] often diverge empirically, likely capturing different constructs (Roisman, 2006; Shaver and Mikulincer, 2002; Strauss et al., 2022). Moreover, studies applying observer-based measures of defenses to the AAI have primarily focused on psychopathology (Békés et al., 2021b; Carlucci et al., 2023; Tanzilli et al., 2021; Tasca et al., 2022), with limited exploration of their relevance to parenting.

In the parenting context, the maternal use of defenses during the recollection of past attachment experiences may have unique implications for the mother–infant relationship. During significant relational transitions (e.g., the birth of a child), a mother’s capacity to effectively manage the physical and emotional demands of parenting is likely to foster more favorable outcomes (e.g., a more positive mother–child relationship, optimal child development) (Perry, 2016). Within this framework, attachment theory predicts that individuals with a secure attachment state of mind will demonstrate unbiased processing of affectively laden information, employing minimal reality-distorting defenses. In contrast, those with insecure or unresolved attachment will be more likely to respond defensively when confronted with negative emotions, threats of separation, or general distress (Cramer and Kelly, 2010; Dykas and Cassidy, 2011).

Empirical research linking attachment mental states to defenses in the parenting context, though limited, has revealed notable patterns. For example, self-reported insecure attachment is typically associated with increased use of immature defenses (e.g., Carone et al., under review; Prunas et al., 2019). Also, among heterosexual pregnant mothers, the use of mature defenses (e.g., humor, altruism) during pregnancy has been associated with children demonstrating greater attachment security, enhanced emotional–social abilities, and fewer behavioral problems (Porcerelli et al., 2016). Although these studies provide key insights into the interplay between attachment and defenses in the parenting context, further research is needed to better understand their implications for the mother–infant relationship.

In this vein, while the *Psychodynamic Diagnostic Manual, second edition* (PDM-2; Lingiardi and McWilliams, 2015, 2017) was not developed as a parenting manual, it provides an integrative framework for understanding the role of attachment and defenses during pregnancy on maternal mental functioning and, subsequently, the mother–infant relationship over time. Of particular relevance to parenting is the PDM-2’s emphasis on defensive functioning as a core component of mental functioning, providing valuable insight into the ways in which parents manage the stresses associated with caregiving. This perspective is crucial in the parenting context, as it highlights how a parent’s mental functioning both shapes and is shaped by interactions with their child. Further proof of the PDM-2’s relevance to maternal functioning and its impact on the mother–infant relationship lies in its detailed focus on the “Infancy and Early Childhood (IEC 0–3)” period. Taking a multiaxial approach, the IEC 0–3 classification addresses key diagnostic components, including functional emotional developmental capacities (Axis II), regulatory-sensory processing capacities (Axis III), relational patterns and disorders (Axis IV), and other medical and neurological diagnoses (Axis V) as determinants of infant disorders (Axis I) (Speranza et al., 2018).

Most relevant to the present study is Axis IV, which is based on evidence deriving from the attachment field (along with those from developmental psychopathology and relational psychoanalysis), and describes and assesses caregiver–infant interactive patterns based on eight key indicators: quality and flexibility of the caregiver’s representation of the infant; quality of the caregiver’s reflective functioning; quality of the nonverbal engagement between caregiver and infant; quality of interactional patterns (reciprocity, synchrony, interactive repair); affective tone of the caregiver–infant relationship; quality of the caregiver’s behavior (sensitivity vs. threatening and/or frightening behaviors); quality of caregiving patterns (comfort, stimulation, responsiveness to the infant’s emotional signals, encouragement vs. withdrawal, overstimulation, controlling behavior, insensitivity); and the infant’s ability to engage and form a significant relationship (vs. specific difficulties that impair this ability).

The present study addressed a critical gap in the literature by conducting a PMD-2–oriented investigation to explore the impact of maternal attachment state of mind and defensive functioning during pregnancy on the mother–infant relationship at 6 months postpartum. While previous research has extensively examined the impact of maternal attachment on mother–infant relationship quality (e.g., Fonagy et al., 1991; Jacobvitz et al., 2025; Lyons-Ruth et al., 1999; Madigan et al., 2015, 2024; van IJzendoorn, 1995), very little research has examined defensive functioning in the context of parenting (Carone et al., under review; Carone and Tracchegiani, 2024; Cramer, 2006; Porcerelli et al., 2016, 2022) and its potential role as a transmission mechanism in this association.

Based on the literature reviewed above, the present study analyzed the direct effect of maternal attachment mental state on mother–infant relationship quality. It hypothesized that greater maternal attachment coherence of mind during pregnancy would predict a healthier mother–infant relationship (Hypothesis 1). Additionally, the study explored the mediating role of maternal defensive functioning while recalling past attachment experiences in this association. It was hypothesized that more adaptive defensive functioning, as a result of greater attachment coherence of mind, would be associated with healthier mother–infant relationships (Hypothesis 2).

## 2 Materials and methods

### 2.1 Participants

A community sample of 68 cisgender heterosexual mothers (*M* = 35.83; *SD* = 4.32) was recruited during their third trimester of pregnancy (time 1 [T1]; *M_gestational weeks_* = 36.30, *SD* = 1.80). Prior to pregnancy, 70.59% (*n* = 48) of the mothers were employed (*n* = 30 full-time, *n* = 18 part-time), while the remaining 29.41% (*n* = 20) were unemployed. Most (*n* = 42, 61.77%) held a high school diploma, while the others had earned a bachelor’s/master’s degree (*n* = 15, 22.06%) or a PhD/specialization (*n* = 9, 13.24%). On average, mothers had 1.75 children (*SD* = 0.71). At time 2 (T2; 6 months postpartum), 58.82% (*n* = 40) of the infants were assigned female at birth, with a mean age of 6.04 months (*SD* = 0.34). Most mothers had a vaginal birth (*n* = 50, 73.53%), while the remainder (*n* = 18, 26.47%) had delivered via Cesarean section. Across both study phases, all mothers were partnered with their child’s father and lived in central Italy (Latium).

### 2.2 Procedure

The Ethics Committee of the Department of Developmental and Social Psychology at Sapienza University of Rome approved the study. Participation was voluntary and confidential, with written informed consent obtained from all mothers. Fathers also provided consent for their child to participate. The principal researcher conducted two home visits for data collection: (1) during the third trimester of pregnancy (T1); and (2) approximately 6 months later (T2). Participants were recruited through maternity care services (*n* = 37, 54.41%), gynecologists (*n* = 18, 26.47%), and word-of-mouth referrals from participating mothers (*n* = 13, 19.12%).

### 2.3 Measures

#### 2.3.1 T1 assessment

##### 2.3.1.1 Maternal attachment state of mind

During the third trimester, mothers completed the Adult Attachment Interview (AAI; George et al., 1985; Main et al., 2002). The AAI is a semi-structured interview comprising questions about childhood relationships with one’s parents, requiring respondents to substantiate their descriptions with specific episodic memories. It also addresses experiences of bereavement and abuse in childhood and adulthood. In the present study, all interviews were audio-recorded, transcribed verbatim, and coded using Main et al.’s (2002) system. Each mother received both a three- and a four-way attachment classification based on their AAI discourse.

A *secure–autonomous* (F) classification is associated with responses that are coherent, clear, relevant, and reasonably succinct. In contrast, a *dismissing* (Ds) classification is characterized by idealization (i.e., overly positive generalizations unsubstantiated by specific memories), an inability to recall specific memories, and/or derogation of attachment figures. A *preoccupied* (E) classification is typically assigned to lengthy, emotionally charged narratives that lack relevance and coherence, with a more passive tone. Finally, an *unresolved* (U) classification applies to transcripts showing signs of disorientation in the discussion of potentially traumatic events (i.e., loss and/or abuse). Respondents with a U classification also receive a secondary classification (i.e., F, Ds, E) that reflects their discourse when not discussing loss or abuse. Relatedly, a fifth AAI category called cannot classify (CC) can emerge as a global breakdown in the organization and maintenance of a singular strategy (Hesse, 1996). Beyond these categorical classifications, the present study used the dimensional score of *coherence of mind*, which is widely regarded as the best index of attachment security/insecurity in adults (e.g., Main et al., 2002; Roisman et al., 2001; Waters et al., 2018).

Interviews were independently coded by two raters who had successfully completed reliability testing. For approximately 25% of a randomly chosen set of AAI transcripts (*n* = 17), interrater agreement for the three- and four-way attachment classifications was excellent, with Cohen’s *κ* = 0.89 and 0.83 (*p* < 0.001), respectively. Disagreements between raters were resolved through discussion, and consensus codes were used for the analyses. Notably, neither AAI rater had been trained on the DMRS-Q and both were blinded to the DMRS-Q scores. Over years, the AAI has become the gold standard assessment of adult attachment research (Bakermans-Kranenburg et al., 2024), and its validity has been demonstrated by a recent meta-analysis involving more than 78 different studies (Verhage et al., 2016).

##### 2.3.1.2 Mothers’ defensive functioning

The Defense Mechanism Rating Scale–Q Sort (DMRS-Q; Di Giuseppe et al., 2014; Di Giuseppe and Perry, 2021)—a computer-based, observer-rated tool for assessing defenses—was applied to the AAI transcripts. The DMRS-Q is based on a gold standard theoretical model (Perry, 1990) and consists of 150 statements that describe 30 defenses, organized into seven hierarchical levels (i.e., action, major image-distorting, disavowal, minor image-distorting, neurotic, obsessional, mature), ranging from least adaptive (i.e., immature) to most adaptive (i.e., mature), in terms of mental states, relational dynamics, verbal and non-verbal expressions, and perceptions of the self and others. Such defenses emerge as responses to internal or external stress or conflict (for an overview, see Di Giuseppe and Perry, 2021).

Although the DMRS-Q is an observer-rated measure, it does not require extensive training or verbatim transcripts of clinical interviews or therapy sessions (Békés et al., 2021a). Rather, a single trained and certified rater sorts the 150 statements, using the DMRS-Q software, into a seven-rank forced distribution that quantifies the contribution of each defense pattern to the respondent’s defensive functioning. The software then generates a comprehensive report that includes: a qualitative description of the respondent’s defensive profile; a so-called *defensive profile narrative* (DPN); and quantitative scores for overall defensive functioning (ODF), seven hierarchically ordered defense levels, and 30 specific defenses. The present study used the ODF score. This score, ranging from 1 (*least adaptive*) to 7 (*most adaptive*), reflects the overall adaptiveness of the respondent’s defensive functioning. Several studies have demonstrated the validity and satisfactory interrater reliability of the DMRS-Q ODF (e.g., Békés et al., 2021a; Di Giuseppe et al., 2014). Approximately 25% of the AAI transcripts (*n* = 17) were double-coded by a second certified rater, yielding an intraclass correlation (ICC) of 0.74 for the ODF (in line with Carlucci et al., 2023). Disagreements between coders were resolved through discussion, and consensus codes were used for the analyses. In the present study, the coders who applied the DMRS-Q were not trained in AAI coding and were blinded to the AAI classifications. The complete DMRS-Q rating procedure is available online, at: https://webapp.dmrs-q.com/.

#### 2.3.2 T2 assessment

##### 2.3.2.1 Mother–infant relationship quality

At 6 months postpartum, mother–infant relationship quality was assessed during 20 min of free-play interaction, which was videotaped for analysis. The videotaped interactions were subsequently coded based on six indicators outlined in the Infant–Caregiver Relationship Scale, Section IV of the Psychodiagnostic Chart—IEC (Lingiardi and McWilliams, 2017, p. 894): (1) quality of the nonverbal engagement between caregiver and infant; quality of interactional patterns (reciprocity, synchrony, interactive repair); affective tone of the caregiver–infant relationship; quality of the caregiver’s behavior (sensitivity vs. threatening and/or frightening behaviors); quality of caregiving patterns (comfort, stimulation, response to infant emotional signals, encouragement vs. withdrawal, overstimulation, controlling behavior, insensitivity); and the infant’s ability to engage and form a significant relationship (vs. specific difficulties that impair this ability).

Additionally, mothers were interviewed about their relationship with their infant, using questions adapted from the Parent Development Interview (PDI; Aber et al., 1985; Slade et al., 2003). Sample questions included: “Could you briefly describe what [infant’s name] is like?” “What do you like most about [infant’s name]?” “What do you like least about [infant’s name]?” and “Could you choose three adjectives that you feel describe the relationship between you and [infant’s name]?” Interviews were subsequently coded for the remaining two indicators of the Infant–Caregiver Relationship Scale (Lingiardi and McWilliams, 2017, p. 894): quality and flexibility of the caregiver’s representation of the infant; and quality of the caregiver’s reflective functioning.

The first author coded each indicator using a 5-point Likert scale ranging from 1 (*severely impaired*) to 5 (*healthy*), and then summed the eight ratings to compute an overall total score for mother–infant relationship quality, with scores ranging from 36–40 (*healthy/adapted relational patterns*) to 8–14 (*major impairments in relational patterns, or relational disorders*) (for a detailed description of these relational patterns, see Lingiardi and McWilliams, 2017, p. 725). To ensure reliability, a master’s student double-coded 25% (*n* = 17) of the video and interview data. Interrater agreement was excellent, with an ICC of 0.82, *p* < 0.001.

### 2.4 Data analysis

**Figure 1:**
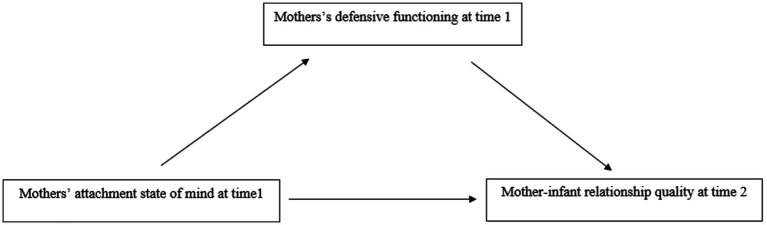
Tested mediation model with mothers’ attachment state of mind at T1 predicting mother-infant relationship quality at T2 through mothers’ defensive functioning at T1 (*N* = 68).

All statistical analyses were conducted using the R software (R Core Team, 2022), with significance set at *p* < 0.05. Descriptive statistics, including means and standard deviations, were calculated for maternal attachment coherence of mind, defensive functioning, and mother–infant relationship quality. Frequencies of mothers’ attachment classifications were also provided, for descriptive reasons. Similarly, Pearson’s bivariate associations between continuous variables were presented. To test the study hypotheses, a mediation model was run using a bootstrap procedure with 95% confidence intervals (CIs) and 5,000 resamples (Hayes, 2017). A *post hoc* Monte Carlo power simulation was computed to estimate the statistical power for detecting the indirect effects, using the shiny and MASS add-on R packages (Schoemann et al., 2017). The tested mediation model is displayed in Figure 1.

## 3 Results

### 3.1 Mothers’ attachment mental states

Considering the AAI three-way distribution, most mothers (*n* = 37, 54.41%) showed a secure-autonomous attachment state of mind. Among the remaining mothers, 18 (26.47%) demonstrated an insecure-dismissing attachment state of mind and 10 (14.71%) showed an insecure-preoccupied attachment state of mind. Three mothers (4.41%) were classified as having a cannot classify state of mind and were excluded from the three-way distribution analysis. When unresolved/cannot classify states of mind were included in the four-way distribution, most mothers (*n* = 36, 52.94%) were still classified as secure-autonomous, while 16 (23.53%) were classified as insecure-dismissing, 6 (8.82%) as insecure-preoccupied, and 10 (14.71%) as unresolved/cannot classify. Both the three-way and four-way distributions aligned with attachment classifications observed in international and national normative samples of mothers (Bakermans-Kranenburg et al., 2024; Cassibba et al., 2013).

### 3.2 Associations between prenatal AAI coherence of mind, defensive functioning, and postnatal mother–infant relationship quality

Pearson’s correlations (Table 1) revealed that higher relationship quality at 6 months postpartum was observed in mother–infant dyads in which mothers exhibited greater coherence of mind [*r*(66) = 0.49, *p* < 0.001] and more adaptive defensive functioning [*r*(66) = 0.43, *p* < 0.001] during the third trimester. Also, maternal coherence of mind and defensive functioning were positively associated [*r*(66) = 0.36, *p* = 0.002].

**Table 1:** Means, standard deviations, and Pearson’s correlations for maternal attachment coherence of mind, defensive functioning, and mother–infant relationship quality (*N* = 68).

Variables	1.	2.	3.	*M*	*SD*
1. T1 AAI coherence of mind	—			5.20	1.92
2. T1 Maternal defensive functioning	0.36^**^	—		5.33	0.67
3. T2 Mother–infant relationship quality	0.49^***^	0.43^***^	—	30.90	6.36

T1 = Time 1 (third trimester). T2 = Time 2 (infant’s 6 months). AAI = Adult Attachment Interview. ^**^p < 0.01. ^***^p < 0.001.

### 3.3 Longitudinal influences of mothers’ prenatal attachment mental states on mother–infant relationship quality through maternal defensive functioning

Mediation analysis, conducted using the bootstrap percentile method with 5,000 resamples, indicated that mothers’ prenatal attachment mental state significantly influenced postnatal mother–infant relationship quality via maternal defensive functioning. Specifically, at 6 months postpartum, mother–infant dyads in which mothers had lower coherence of mind during the third trimester exhibited less healthy relationship quality, mediated by less adaptive maternal defensive functioning. Additionally, the direct effect of maternal coherence of mind on mother–infant relationship quality was significant. The overall model accounted for 31% of the variance. Full statistical details are presented in Table 2. A Monte Carlo power analysis of the indirect effects demonstrated a moderate power of 64% (based on 95% confidence intervals; Schoemann et al., 2017).

**Table 2:** Longitudinal influences of AAI coherence on mind on mother–infant relationship quality through maternal defensive functioning (*N* = 68).

Type	Effect	Estimate	SE	95% C.I.		β	*p*
				Lower	Upper		
Indirect	T1 AAI coherence of mind ⇒ T1 maternal defensive functioning ⇒ T2 mother–infant relationship quality	0.35	0.17	0.03	0.74	0.11	0.040
Component	T1 AAI coherence of mind ⇒ T1 maternal defensive functioning	0.13	0.04	0.04	0.21	0.36	0.001
	T1 maternal defensive functioning ⇒ T2 mother–infant relationship quality	2.76	1.03	0.36	4.78	0.29	0.008
Direct	T1 AAI coherence of mind ⇒ T2 mother–infant relationship quality	1.28	0.36	0.52	2.07	0.39	<0.001
Total	T1 AAI coherence of mind ⇒ T2 mother–infant relationship quality	1.63	0.35	0.79	2.40	0.49	<0.001

T1 = Time 1 (third trimester). T2 = Time 2 (infant’s 6 months). AAI = Adult Attachment Interview. C.I. = Confidence Intervals.

## 4 Discussion

The present findings enhance our understanding of how maternal attachment mental states and defensive functioning during pregnancy shape the quality of the mother–infant relationship during the early postnatal period. Consistent with our hypotheses, greater maternal coherence of mind predicted healthier mother–infant relationships. Furthermore, defensive functioning mediated this relationship, such that greater coherence of mind predicted more adaptive defensive functioning, resulting in healthier mother–infant interactions at 6 months postpartum.

Concerning the first hypothesis, the findings provide empirical support for previous research highlighting the role of attachment coherence of mind in caregiving (e.g., Carone et al., 2024; Main et al., 2002; Waters et al., 2018). Mothers with coherent narratives of their attachment histories were more likely to exhibit caregiving patterns that fostered secure, responsive, and mutually satisfying interactions with their infants. This finding supports the central tenet of attachment theory, which posits that maternal internal working models shape the ways in which mothers interpret and respond to relational cues (Bowlby, 1980; George and Solomon, 2008).

However, our second hypothesis extended this understanding by highlighting the mediating role of defensive functioning. Specifically, the ways in which mothers defended against emotional distress while recalling their own attachment experiences emerged as a critical factor in shaping their caregiving behaviors. Mothers with higher overall defensive functioning—indicative of the use of more mature defenses—were better able to manage the emotional intensity of reflecting on their past attachment experiences. This capacity allowed them to approach caregiving with greater emotional availability and attunement. Conversely, maladaptive defenses appeared to hinder mothers’ capacity to process their own attachment histories, leading to caregiving patterns marked by emotional withdrawal, intrusiveness, or unresponsiveness.

This dynamic underscores the significance of maternal defenses as a bridge between mothers’ own childhood experiences and their current caregiving behaviors. Reflecting on past attachment relationships may evoke feelings of vulnerability, particularly in mothers grappling with unresolved traumas, losses, or unmet emotional needs from childhood. Adaptive defenses enable mothers to manage these emotional challenges constructively, allowing them to prioritize their infant’s needs (Perry, 2016) without becoming overwhelmed by their own unresolved states of mind. Conversely, maladaptive defenses may impede this process by heightening emotional reactivity, numbing emotional engagement, or distorting perceptions of the infant’s needs (Perry, 2016). For example, a mother relying on projection may misinterpret her infant’s cues as evidence of rejection or hostility, resulting in inconsistent or intrusive caregiving behaviors. Similarly, a mother who employs denial may struggle to recognize and address her infant’s emotional needs, resulting in relational misattunement. While these findings echo psychodynamic theories and clinical observations (Carone, 2025; Perry, 2016), future research is needed to provide empirical evidence.

Of note, the mediating role of defensive functioning in the relationship between maternal attachment coherence of mind and mother–infant relationship quality advances the understanding of psychodynamic processes in parenting. Specifically, it highlights the role of unconscious defenses in regulating maternal responses to stressors, particularly during the perinatal period. While prior research has established associations between self-reported insecure attachment styles and maladaptive defenses (Mikulincer et al., 2009; Prunas et al., 2019), few studies have explored these constructs in the context of early caregiving (Carone et al., under review) while employing gold-standard measures of attachment (e.g., AAI) and defenses (e.g., DMRS-Q). The present study’s findings align with Porcerelli et al. (2016), who demonstrated that using mature defenses during pregnancy predicted positive relational outcomes in toddlerhood. By extending this line of inquiry to the 6-month postpartum period, the present results underscore the continuity of maternal mental functioning from pregnancy to early caregiving.

However, while the indirect effect was statistically significant, it appeared small. This suggests that other mechanisms may have a greater influence on the association between prenatal maternal attachment state of mind and postnatal mother-infant relationship quality, including potential shifts in mothers’ attachment after childbirth. This possibility is particularly relevant given evidence that the transition to parenthood provides a crucial opportunity for mothers to reflect on their own experiences of being parented as they make decisions about raising their own children (George and Solomon, 2008).

Becoming an infant’s primary attachment figure may influence a mother’s internal working model of attachment by offering new insights into what it means to be both an attachment figure and a parent. Parenthood, therefore, may serve as a catalyst for reevaluating past relationships (George and Solomon, 2008), potentially shaping mothers’ understanding of how and why others behave as they do—including their own parents’ caregiving behavior. Such changes may be especially pronounced for mothers with an insecure or unresolved attachment state of mind, which tend to be less stable than a secure-autonomous state of mind (e.g., Bakermans-Kranenburg and van IJzendoorn, 1993; Benoit and Parker, 1994; Sagi et al., 1993; Spieker et al., 2011). Future research with larger samples could provide further insight into these dynamics. Additionally, the shared variance resulting from the use of both the AAI and the DMRS-Q on the same interview transcript may have contributed to the small effect size of the indirect effect.

Overall, the findings corroborate prior research emphasizing the role of defenses in regulating relational stress and processing emotionally charged experiences (Mikulincer et al., 2009; Vaillant, 1992), such as caregiving. They also affirm the utility of the PDM-2 (Lingiardi and McWilliams, 2015, 2017) as a comprehensive framework for understanding maternal mental functioning and its implications for caregiving and mother–infant relationships, especially during critical developmental periods such as pregnancy and the early postpartum phase. While the PDM-2 was not explicitly developed for parenting assessments, it underscores the centrality of defensive functioning as a core aspect of mental functioning. Additionally, its hierarchical organization of defenses, ranging from immature to mature, underscores the relevance of defenses in managing both intrapsychic and interpersonal challenges (Perry, 1990; Vaillant, 1977, 1992). Similarly, as highlighted in the IEC section of the PDM-2 (Speranza et al., 2018), maternal mental functioning significantly influences the quality of interactions with infants, particularly during the early postpartum period. Therefore, the present findings underscore the importance of mature defenses in fostering relational health, as mothers with greater attachment coherence of mind and more adaptive defensive functioning were better equipped to engage in synchronous and emotionally responsive interactions with their infants (Perry, 2016).

### 4.1 Limitations, strengths, and future directions

Despite the contributions of the study, several limitations must be acknowledged. First, the relatively small sample size limited statistical power, particularly for detecting smaller effect sizes. Future research should attempt to replicate the findings in larger and more diverse samples, to increase generalizability. Such studies may also enable a more detailed examination of whether different attachment mental states are associated with distinct defensive profiles and how these variations impact mother–infant relationship quality.

Second, the study focused exclusively on cisgender heterosexual mothers from a single cultural context, thereby limiting the applicability of the findings. As cultural norms and gender dynamics may influence caregiving practices and defensive processes (Carone et al., under review), future research should include more diverse parent populations to explore these dimensions further. Third, mothers’ attachment state of mind at T2 was not assessed. Therefore, it cannot be ruled out the possibility that maternal attachment changed between pregnancy and the postpartum period, as suggested by previous research (Bakermans-Kranenburg and van IJzendoorn, 1993; Benoit and Parker, 1994; Sagi et al., 1993; Spieker et al., 2011). Finally, while the study elucidated the relationship between maternal attachment mental states, defenses, and mother–infant relationships, it did not account for factors such as infant temperament or environmental influences (e.g., partner support) (e.g., Takács et al., 2020; Zhu et al., 2024), which are known to shape mother–infant relationship quality. Future research incorporating these variables could provide a more comprehensive understanding of the interplay between maternal attachment, defenses, and caregiving behaviors.

Notwithstanding these limitations, the study demonstrates several methodological and theoretical strengths. First, the use of the AAI and DMRS-Q allowed for an in-depth assessment of maternal attachment mental states and defenses while using gold standard measures of both constructs. Unlike self-report measures, these observer-rated tools minimize bias and capture unconscious processes, thereby enhancing the validity of the findings. Relatedly, the Infant–Caregiver Relationship Scale was developed within the PDM-2 framework (Lingiardi and McWilliams, 2017), drawing on both empirical evidence and clinical experience. Its application to 20 min of free-play interaction and a semi-structured interview ensured the collection of sufficient details on the various dimensions characteristic of the mother-infant relationship.

In this vein, the study may advance the parenting and attachment fields by highlighting the utility of observer-rated methods such as the DMRS-Q in capturing the unconscious processes underlying maternal attachment. Of further note, the use of the AAI not only to assess mothers’ attachment mental states but also as a stimulus to activate mothers’ attachment systems (e.g., Dozier and Kobak, 1992; Farina et al., 2014) enabled an investigation of whether maternal attachment coherence of mind determined the quality of mother–infant interactions not only directly, but also through the ability to process stressful experiences (i.e., painful attachment memories). However, further studies applying the DMRS-Q to the parents’ AAI are needed to confirm its reliability in the parenting context.

Second, the longitudinal design enabled the examination of prenatal factors influencing postnatal outcomes, thereby contributing to the literature on the intergenerational transmission of attachment and its effects on parenting (Grienenberger et al., 2005; Shlafer et al., 2015; van IJzendoorn, 1992). Finally, the focus on defensive functioning as a mediator addressed a critical gap in the literature, highlighting the relevance of this factor for mother–infant relationship quality. This evidence has been well received by the editors of the PDM, who have included an appendix on the assessment of mental functioning in the parenting context in the forthcoming third edition (Carone, 2025).

### 4.2 Clinical implications

The present findings have significant implications for parenting interventions during the perinatal period. Screening for maternal attachment states of mind and mental functioning during pregnancy could help identify at-risk mothers and inform tailored interventions aimed at enhancing mother–infant relationships. Additionally, the incorporation of strategies to strengthen adaptive defenses may bolster mothers’ emotional resilience during the early postpartum period. Psychodynamic interventions that focus on increasing awareness and flexibility in defensive functioning, as outlined in the PDM-2 framework (Lingiardi and McWilliams, 2015, 2017), could support mothers in managing caregiving stressors more effectively. These approaches underscore the need for holistic assessments and interventions that address both attachment and defensive processes as integral components of maternal mental functioning.

### 4.3 Conclusion

The present study highlights the critical role played by maternal defenses during the recollection of personal attachment histories in shaping early caregiving behaviors. Future research should continue to explore the complex dynamics between maternal functioning and mother–infant relationships, incorporating diverse populations and broader contextual factors to deepen our understanding of early relational health. Clinical interventions that address maternal attachment coherence and promote defensive functioning hold promise for fostering secure infant–mother bonds and promoting positive developmental outcomes.

## Data Availability

The datasets presented in this article are not readily available because the datasets contain sensitive data which cannot be anonymized. Requests to access the datasets should be directed to Nicola Carone, nicola.carone@uniroma2.it.
